# MicroRNA-148a overexpression improves the early development of porcine somatic cell nuclear transfer embryos

**DOI:** 10.1371/journal.pone.0180535

**Published:** 2017-06-30

**Authors:** Ping Wang, Xiangping Li, Lihua Cao, Shihai Huang, Haiyan Li, Yan Zhang, Ting Yang, Jianrong Jiang, Deshun Shi

**Affiliations:** 1 State Key Laboratory for Conservation and Utilization of Subtropical Agro-bioresources, Guangxi University, Nanning, Guangxi Zhuang Autonomous Region, China; 2 College of Life Science and Technology, Guangxi University, Nanning, Guangxi Zhuang Autonomous Region, China; University of Texas at Austin Dell Medical School, UNITED STATES

## Abstract

Incomplete epigenetic reprogramming of donor cell nuclei is one of the main contributors to the low efficiency of somatic cell nuclear transfer (SCNT). To improve the success of SCNT, somatic cell DNA methylation levels must be reduced to those levels found in totipotent embryonic cells. Recent studies have demonstrated that miR-148a can affect DNA methylation via *DNMT1* modulation in various cancers. Therefore, the focus of this study was to examine the influence of miR-148a on DNA methylation in donor cells and in SCNT embryo development. Thus, a stable cell line overexpressing miR-148a was established and used to produce SCNT embryos. Upon examination, *DNMT1* was found to be a miR-148a target in porcine fetal fibroblasts (PFF). Furthermore, miR-148a overexpression in PFFs significantly decreased *DNMT1* expression and global DNA methylation levels (*P < 0*.*05*). Moreover, miRNA-148a expression levels in SCNT embryos were significantly lower at the 2-cell and 4-cell stages when compared to IVF and parthenogenetic embryos. The group overexpressing miRNA-148a also showed a significant increase in blastocyst formation and total cell numbers (*P < 0*.*05*). Additionally, miR-148a overexpression altered the immunofluorescence signal of 5-mC and H3K9ac, and enhanced pluripotent gene (*Oct4* and *Nanog*) expression levels during embryo development. These results indicate that miR-148a overexpression enhances the developmental potential of SCNT embryos and modifies epigenetic status.

## Introduction

Since the birth of the first cloned animal, Dolly, in 1996, somatic cell nuclear transfer (SCNT) has been successful in a variety of mammalian species [1]. However SCNT technology is still inefficient due to a variety of issues such as low cloning efficiency, fetal abnormalities, and placental deficiency [2]. One of the major causes of SCNT inefficiency is incomplete epigenetic reprogramming of the donor cell nuclei, which subsequently leads to aberrant gene expression during embryo development [3].

Chromosomal DNA methylation is an important epigenetic modification that plays a significant regulatory role in gene transcription [4–6]. In SCNT embryos, DNA hypermethylation levels are similar to those of somatic cells due to aberrant DNA methyltransferases (DNMTs) expression [3, 7, 8]. Of these DNMTs, *DNMT1* has been suggested to contribute to the aberrant methylation status in SCNT embryos. Therefore, various approaches have been used to inhibit donor cell *DNMT1* expression, thereby promoting somatic cell nuclear reprogramming and cloning efficiency. These approaches include utilizing hypomethylating drugs (5-aza-dC, RG108), gene knockout, or siRNA [9–12].

MicroRNAs (miRNAs) are short non-coding regulatory RNA molecules that inhibit translation or contribute to mRNA degradation via binding to the 3′UTR of target mRNAs [13]. MiRNAs are involved in many biological processes during early embryonic development, including proliferation, differentiation, and gene expression regulation [14]. MiRNAs and DNA methylation are both critical regulators of gene expression and have been shown to exhibit an interconnected relationship in cancers. For example, DNA methylation can inhibit miRNA transcription by methylating CpG islands within miRNA promoter regions [15]. Moreover, multiple miRNAs directly target DNMTs, with *DNMT3a* and *DNMT3b* directly targeted by miR-29b and *DNMT1* by miR-342 and miR-148a/152 [15–19]. In gastric cancer, aberrant DNA methylation of CpG islands neighboring miR-148a results in miR-148a silencing and subsequent DNMT1 overexpression [20]. While miR-148a expression can be restored following 5-aza-dC treatment, conversely its expression was significantly up-regulated after DNMT1 knockdown [15]. Additionally, miR-148a modulation of DNMT1 can be seen in systemic lupus erythematosus, human malignant cholangiocytes and CD^4+^ T cells [21]. While the alteration of miR-148a expression has been used to modulate *DNMT1* in many cancers, to our knowledge, it has not been utilized in donor cells.

The aim of this study is to examine the function of miR-148a in the early development of porcine SCNT embryos. Herein, *DNMT1* was confirmed to be a miR-148a target in porcine fetal fibroblasts (PFF) following luciferase analysis. Additionally, a stable cell line overexpressing miR-148a was established herein and demonstrated that miR-148a overexpression directly represses *DNMT1* and reduces global DNA methylation levels. This stable cell line was subsequently utilized to generate SCNT embryos that showed increased blastocyst formation and total cell numbers, altered immunofluorescent signaling for 5-mC and H3K9ac, and an enhanced expression of pluripotent genes. The results presented herein indicate that miR-148a enhances the developmental potential of SCNT embryos and modifies their epigenetic status.

## Materials and methods

### Animal ethics

All experiments and protocols were performed in strict accordance with the Guide for Care and Use of Laboratory Animals and explicitly approved by the Guangxi University Committee on Animal Research and Bioethics.

### Preparation of porcine fetal fibroblast cells

PFFs were obtained from the ear skin of a newborn Guangxi Debao black piglet. The tissue was stored in Dulbecco’s Phosphate Buffered Saline (DPBS) modified and transported to the laboratory within 8 h. Primary cells were obtained using a tissue block method as previously described [22]. Briefly, the obtained tissue was cut into 2–3 mm^2^ pieces. The disaggregated cells were incubated in a 60-mm culture dish with Dulbecco Modified Eagle medium (DMEM; Gibco) containing penicillin (100 IU/ml; Gibco), streptomycin (100 mg/ml; Gibco), and 10% (v/v) fetal bovine serum (FBS; Gibco) and incubated in a humidified atmosphere of 5% CO_2_ at 38.5℃. After 3 to 7 d in culture, fibroblast cell monolayers formed and were cultured to 80% or 90% confluency. The confluent cells were then digested in 0.25% trypsin with 0.05% EDTA for 3 min, resuspended in freezing medium consisting of 8% dimethyl-sulfoxide (DMSO; Sigma) and 20% FBS in DMEM, and stored in liquid nitrogen until further use.

### Luciferase reporter assay

The 3′UTR of the *DNMT1* gene, which contains a miR-148a target site, was cloned into a pEZX-MT05 vector (GeneCopoeia) containing SV40 promoter-driven Gaussia luciferase and CMV promoter-driven secreted alkaline phosphatase (SEAP) genes. A mutant DNMT1 3′UTR was also constructed. 293T cells were seeded in 24-well plates for 1 day and then co-transfected with pEZX-miR-148a or pEZX-miR-control and pEZX-DNMT1-3′UTR-WT or transfected with pEZX-DNMT1-3'UTR-MUT (3:1) using Lipofectamine LTX and PLUS^TM^ (Invitrogen). At 48 h post-transfection, the supernatants were collected and the luciferase activities were measured on a Centro LB 960 microplate luminometer (Berthold Technologies) using a Secrete-Pair Dual Luminescence Assay kit (GeneCopoeia) according to the manufacturer’s protocols.

### Establishment of cell lines

Lentivirus were packaged as previously described [23]. Briefly, pEZX-miR-148a or pEZX-miR-con (control) lentiviral vectors (GeneCopoeia) and 2 packaging plasmids (NRF, VSVG) were co-transfected into 293T cells using lipofectin. After transfection 48–72 h, the supernatant was collected, filtered with a 0.45-μm filter and the lentiviral titer was determined via serial dilution. The second generation of PFFs were seeded in 60-mm dishes, cultured to 50% confluency and infected with 50 MOI viral solution and 8 μg/mL polybrene. After incubation 24 h, the viral solution was replaced with fresh complete medium of for another 48 h. The positive transgenic resistant clones were obtained by adding 4μg/mL puromycin into the culture medium every 2 to 3 days. After nontransfected cells were killed off, the positive transgenic cells were cultured in fresh medium with 2μg/mL of puromycin added for an additional month, and used for further experiments.

### Oocyte collection and *in vitro* maturation

Porcine ovaries were obtained from local slaughterhouses and transported to the laboratory in a thermos containing PBS at 35–39°C within 4 h of collection. Cumulus-oocyte complexes (COCs) were aspirated from follicles with diameters of 3 to 6 mm using a 10-ml disposable syringe with an 12-gauge needle. Only COCs with uniform cytoplasms and at least 3 layers of compact cumulus cells were washed 3 times with cell culture medium (CCM) containing 2–3% FBS. Sets of 30 COCs were matured in 150-μl droplets of TCM-199 containing 10% porcine follicular fluid, 0.6 mM cysteine, 50 ng/ml epidermal growth factor (EGF), 10 ng/ml IGF-I, 15 IU/ml eCG and 10 IU/ml hCG for 22 h. COCs were further cultured in fresh maturation medium without eCG and hCG for another 20 h at 38.5°C and 5% CO_2_. The matured COCs were denuded using 0.1% hyaluronidase and oocytes with polar bodies were selected for further experiments.

### Somatic cell nuclear transfer

Denuded metaphase II (MII) oocytes that had extruded the first polar body were selected for enucleation in a single microdrop of 150 μL HEPES-M199 medium containing 7.5 μg/mL cytochalasin B using a 25-mm micropipette and an inverted microscope. The donor cells were transferred into the perivitelline space of the enucleated oocytes with a 25-mm micropipette and the oocyte-donor cell couplets were transferred into PZM3 medium and incubated at 38.5°C and 5% CO_2_ for 1 h prior to electrical fusion treatment. The couplets were equilibrated in fusion medium (0.28 M mannitol, 0.5 mM HEPES, 0.01% BSA, 1 mM CaCl_2_, and 0.1 mM MgCl_2_) for 1–2 min, transferred into a chamber with platinum wire electrodes (1 mm apart), and activated with an electric stimulus consisting of single pulses of 1.2 kv/cm for 30 μs using a BTX manipulator 2000 (BTX, San Diego, CA). After 1 h, the couplets without a donor cell in the perivitelline space were considered reconstructed oocytes and were cultured in PZM3. Cleavage and blastocyst formation were subsequently observed under a stereomicroscope on days 2 and 6 respectively. At 168 h, blastocysts were fixed in 4% formaldehyde overnight and stained with 10 mg/mL 4', 6-diamidino-2-phenylindole (DAPI; Sigma) for 10 min. The samples were then mounted and total cell numbers were assessed under an epifluorescence microscope (Nikon).

### *In vitro* fertilization (IVF)

Twenty denuded MII oocytes were placed in 50-μl drops of modified tris-buffered medium (mTBM) containing 2 mM caffeine. Semen was supplied by a pig farm and stored at 17°C for up to 4 d. The sperm pellet was prepared using a Percoll density gradient, with the obtained sperm resuspended in mTBM. MII oocytes were inseminated with a final concentration of 1×10^5^ sperm/ml and incubated at 38.5°C and 5% CO_2_. After 5 h of co-incubation, the gametes were cultured in PZM3.

### Parthenogenetic embryo production

Denuded MII oocytes were equilibrated for 1–2 min in electroporation medium (300 mM mannitol, 0.1 mM CaCl_2_, 0.1 mM MgSO_4_, 0.5 mM HEPES, and 0.01 mg/mL BSA), transferred into a chamber with platinum wire electrodes (1 mm apart), and activated with an electric stimulus consisting of single pulses of 1.2 kv/cm for 30 μsec using a BTX manipulator 2000 (BTX, San Diego, CA). The activated oocytes were washed 3 times in PZM3 and cultured in 30 mL microdrops (10–15 oocytes per drop) of PZM3 under mineral oil at 38.5°C and 5% CO_2_.

### RNA extraction and quantitative real-time PCR

Total RNA was extracted from PFF cells using TRIzol reagent (Ambion, Inc.). The first-strand cDNA was generated using a Prime Script 1st strand cDNA synthesis kit (Takara, Japan) according to the manufacturer’s protocols. Five embryos were collected at each stage (2-cells, 4-cells, or blastocyst) and cDNA was obtained using a Cells-to-cDNA Ⅱ kit (Ambion Inc, Australia). The stem-loop reverse transcription primers for mature miR-148a and U6 were used to detect miRNA expression (Table 1). The obtained cDNA was stored at -80°C until further use.

**Table 1:** List of primers used for reverse transcription and polymerase chain reaction.

primer name	sequence 5'-3'
Reverse transcription primers	
Loop-RT-miR-148a	5'-GTCGTATCCAGTGCAGGGTCCGAGGTATTCGCACTGGATACGACACAAAG-3'
RT-*U6*	5'-CGCTTCACGAATTTGCGTGTCAT-3'
Polymerase chain reaction primers	
*U6*	F 5'-CTCGCTTCGGCAGCACA-3'
	R 5'-AACGCTTCACGAATTTGCGT-3'
miR-148a	F 5'-GGTCAGTGCACTACAGAACTTT-3'
	R 5'-GTGCAGGGTCCGAGGT-3'
*DNMT1*	F 5'-CAGACAAGTGGTGAGGAC-3'
	R 5'-TGTGAAATGAGATGTGATGG-3'
*DNMT3a*	F 5'-TACCCGGGATGAACAGGTCT-3'
	R 5'-TGGACACGTCTGTGTAGTGC-3'
*Oct4*	F 5'-TCAGCCAAACGACCATCT-3'
	R 5'-CTTCCTCCACCCACTTCT-3'
*Nanog*	F 5'-TCCTCTTCCTTCCTCCAT-3'
	R 5'-TCCTTCTCTGTGCTCTTC-3'
*β-ACTIN*	F 5'-GGACCTGACCGACTACCTCA-3'
	R 5'-CCATCTCCTGCTCGAAGTCC-3'

Quantitative real-time PCR (qRT-PCR) was performed using a SYBR PrimeScript RT-PCR kit (Takara, Dalian, China) according to the manufacturer’s instruction. The 20 μL PCR mixture contained 2 μL reverse-transcription product, 10 μL SYBR Green mix, 0.4 μL Rox, 7 μL RNase-free water, 0.3 μL forward, and 0.3 μL reverse primers (Table 1). The amplification reaction was performed at 95°C for 10 min initially, followed by 40 cycles at 30 s at 95°C and 60°C for 1 min. U6 RNA was used as an endogenous reference for miR-148a expression and relative expression levels were calculated using the 2-^ΔΔ^Ct method.

### Western blot analysis

Cells were collected and lysed with RIPA buffer for 30 min on ice, centrifuged at 12,000 g for 30 min, and the supernatants were collected. The proteins were separated via SDS polyacrylamide gel electrophoresis (SDS-PAGE) and transferred to a nitrocellulose filter membrane. The membranes were blocked with 5% skimmed milk in TBST (50 mM Tris, 150 mM NaCl, and 0.1% Tween) for 1 h at room temperature (RT). The membranes were then incubated overnight at 4°C with mouse monoclonal anti-DNMT1 antibodies (Novus; 1:1000) or mouse monoclonal anti-b-actin antibodies (1:2000). Membranes were then washed 3 times for 10 min in TBST and then incubated for 1 h at RT with peroxidase-conjugated goat anti-mouse IgG (Protein Tech Group, Inc.; 1:3000). Membranes were then washed 3 times in TBST, visualized using enhanced chemiluminescence (ECL), and imaged using the ChemiDoc System (Bio-Rad).

### Immunohistochemistry analysis

PFFs and positive transgenic cells that had been seeded on cover glasses were fixed with 4% PFA in PBS for 30 min at RT. Embryos at the 2-cell, 4-cell, and blastocyst stages were selected for fixation. After washing 3 times in PBS containing 0.01% Triton X-100 and 0.3% BSA (TBP), the samples were permeabilized in 1% Triton X-100 in PBS for 30 min and blocked with 1% BSA in PBS for 1 h. After washing 3 times in TBP, the samples were incubated with mouse monoclonal anti-5-methylcytosine antibodies (Millipore; 1:100) or rabbit polyclonal anti-H3K9ac antibodies (Abcam; 1:200) overnight at 4°C. Following incubation, samples were washed in TBP, incubated with anti-mouse or anti-rabbit fluorescein isothiocyanate-conjugated IgG antibodies (Milipore; 1:200) for 1 h at RT, and then washed 3 times in TBP. The samples were then counterstained with 10 mg/mL 4', 6-diamidino-2-phenylindole (DAPI; Sigma) for 10 min at RT. Lastly, the samples were washed in TBP, the cover glasses were mounted onto slides with a drop of mounting medium (Fluoromount-G; SouthernBiotech, USA), and the samples were observed under a laser scanning confocal microscope (Zeiss LSM510 Meta).

### Statistical analysis

Each experiment was performed with at least 3 replicates. The SCNT embryos that underwent cleavage and the development to the blastocyst stage were analyzed by Student’s t tests using the SPSS 17.0 program for Windows. Gene expression levels and DNA methylation patterns between different groups were analyzed by one-way ANOVA using SPSS 17.0. All experimental data are displayed as a mean ± standard error of 3 independent observations, with *P* < 0.05 considered statistically significant.

## Results

### *DNMT1* is the direct target of miR-148a

**Fig 1:**
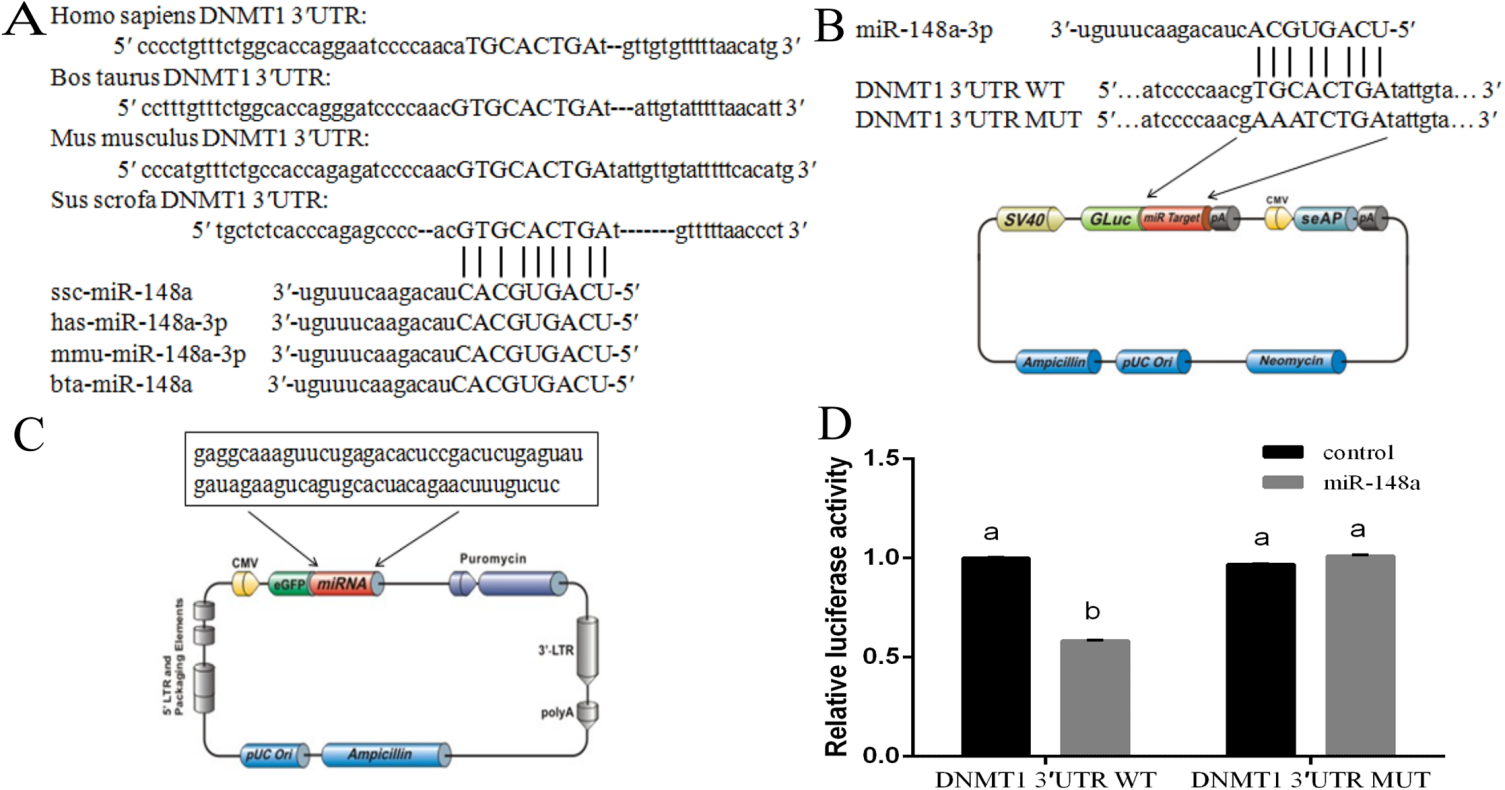
DNMT1 was target gene of miR-148a. (A) miR-148a is highly complementary to pig DNMT1 3'UTR. This potential miR-148a target site is conserved in human, cows, mice and pigs. (B) Schema contains WT and Mut 3-′UTRs of DNMT1, indicating the interaction sites between miR-148a and 3-′UTR of DNMT1. (C) Schema of miR-148a precursor sequence in pEZX-MR03. (D) Luciferase activities were measured in 293T cells co-transfected with the pEZX-DNMT1 (wild type or mutant) and pEZX-miR-148a or pEZX-miR-control. The data were reported as mean ± S.D. for three independent experiments (*p* < 0.05).

*DNMT1* was predicted to be a miR-148a target following TargetScan, miRanda, and DIANA-microT online software analysis. Furthermore, the examined *DNMT1* 3′UTR element was found to be partially complementary to human, cattle, mice, and pig miR-148a (Fig 1A), thus suggesting that miR-148a is highly conserved among various species. To examine the effect of miR-148a on *DNMT1*, a pEZX-miR-148a plasmid was inserted has-miR-148a, which over-expresses miR-148a, and a pEZX-MT05 plasmid was inserted *DNMT1* 3′UTR, with or without mutations, which named pEZX-DNMT1-3′UTR-WT or pEZX-DNMT1-3′UTR-MUT (Fig 1B and 1C). The effect of miR-148a was then observed by co-transfecting 293T cells, with significantly reduced luciferase activity noted in the WT construct relative to the negative control (*P* < 0.05; Fig 1D). In contrast, no obvious change in the mutant 3′UTR reporter plasmid expression was observed.

### Effects of miR-148a over-expression on DNA methylation in PFF cells

**Fig 2:**
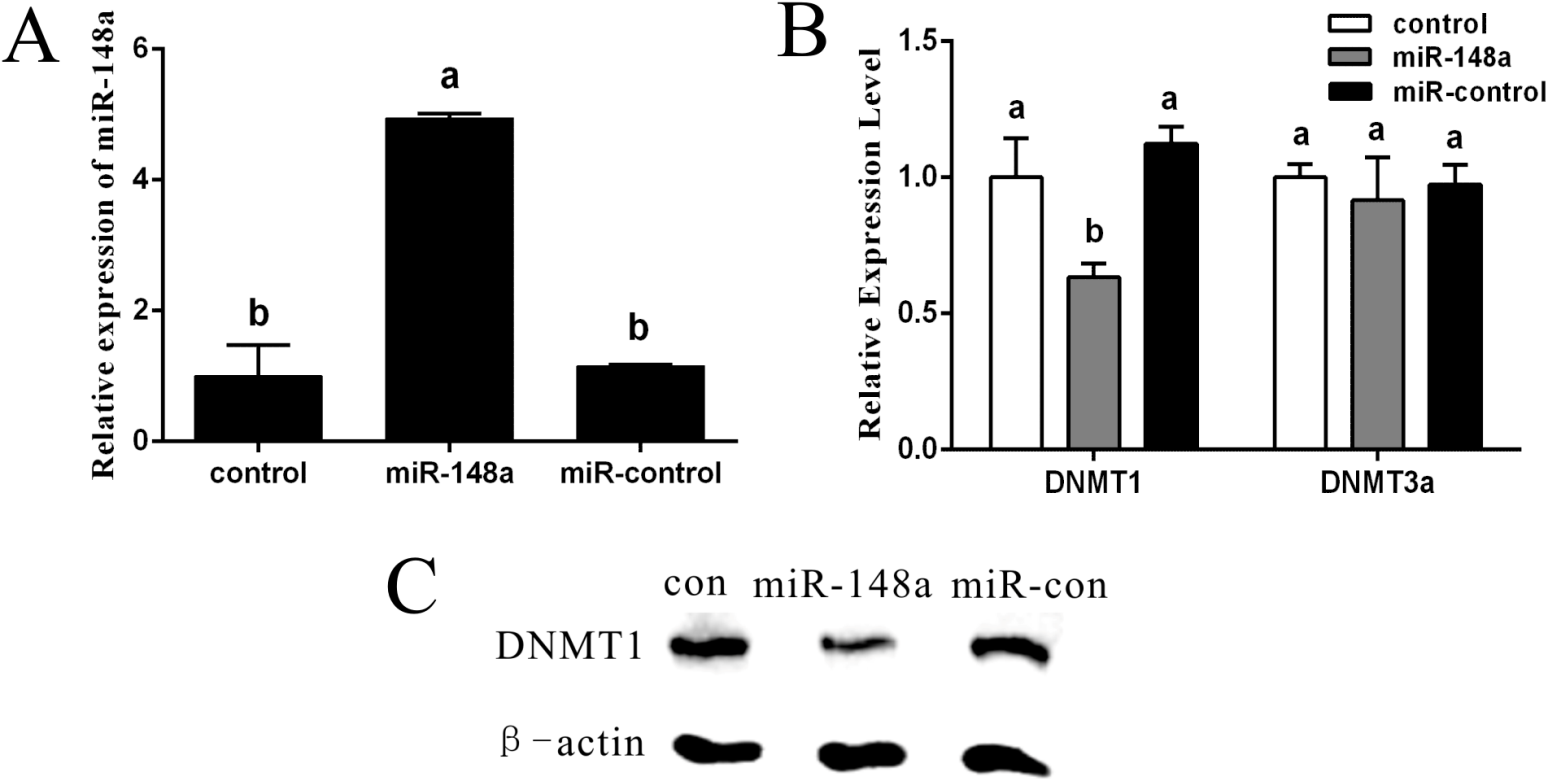
miR-148a inhibited DNMT1expression. (A) miR-148a expression was increased in PFFs of overexpressing miRNA-148a. (B) DNMT1 expression was decreased in PFFs of overexpressing miRNA-148a. DNMT3a expression has no difference in PFFs of overexpressing miRNA-148a compared with control group. (C) Western blot assays confirmed that DNMT1 expression were downregulated by in PFFs of over-expression miR-148a. Values with different superscript letters (a, b) within groups indicate significant difference (*p* < 0.05).

**Fig 3:**
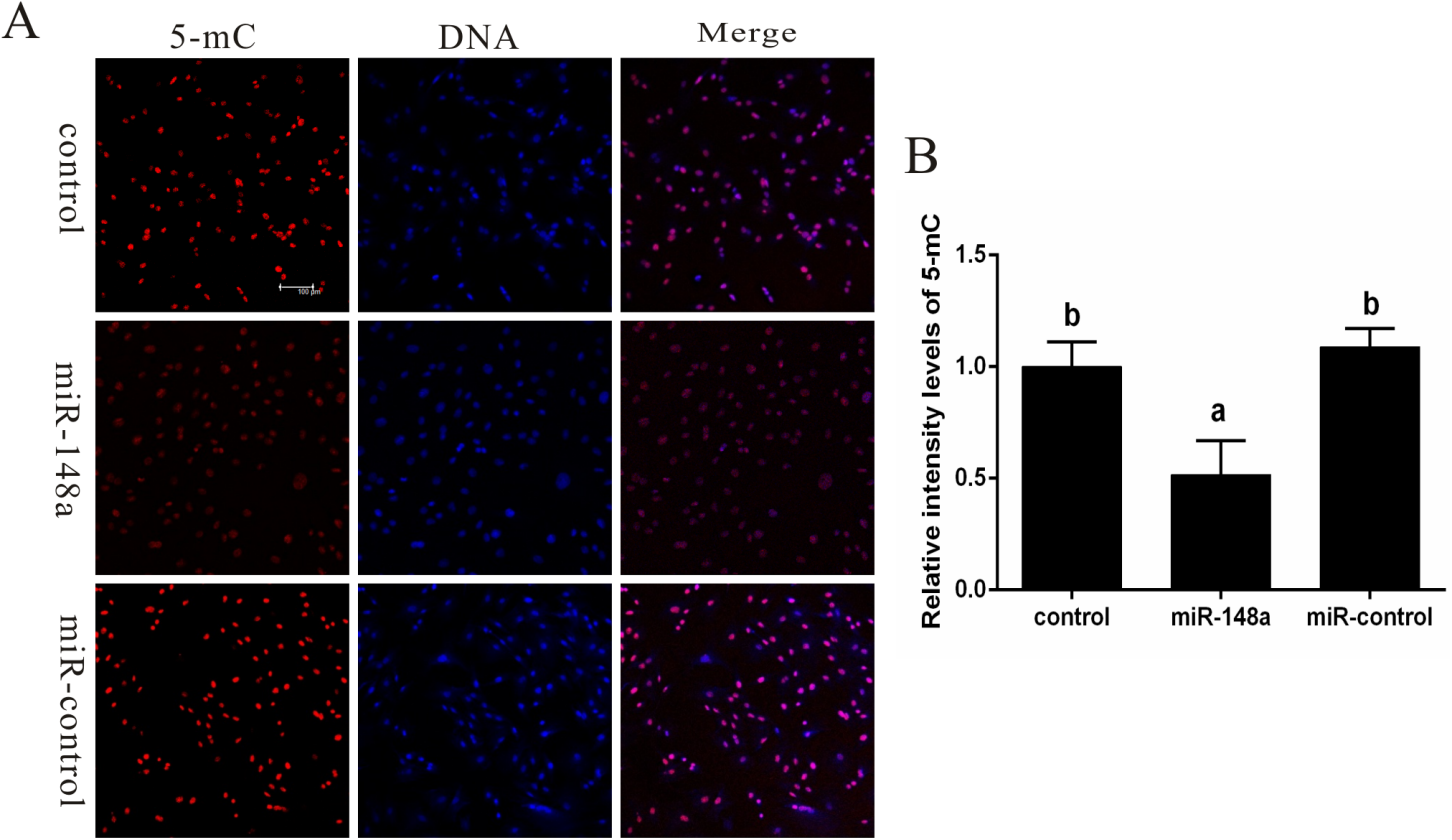
Effect of miR-148a on DNA methylation in PFFs. (A) Methylation patterns were determination on the basis of the relative levels of 5-methyl-cytosine (5-mC) in PFFs. Each sample was counter-stained with DAPI to visualize DNA. (B) Average optical intensity of global DNA methylation measured using Image-Pro Plus 6.0 software. Values are presented as mean ± SEM. Values with different superscript letters (a, b) within groups indicate significant difference (*p* < 0.05). Scare bar = 100μm.

While miR-148a up-regulation can subsequently down-regulate *DNMT1* in cancer cells, it has yet to be examined in PFF cells. Therefore, the effect of miR-148a up-regulation on *DNMT1* expression and DNA methylation patterns was examined in PFF cells. The results showed that in PFF cells overexpressing miR-148a, miR-148a expression increased 4.9-fold (*P* < 0.05; Fig 2A) which resulted in a reduction of *DNMT1* mRNA (*P* < 0.05; Fig 2B) and protein (Fig 2C), but did not affect *DNMT3a* mRNA (Fig 2B). Furthermore, DNA methylation levels were significantly reduced relative to the control cells (*P* < 0.05; Fig 3).

### Characterization of miR-148a expression in SCNT

**Fig 4:**
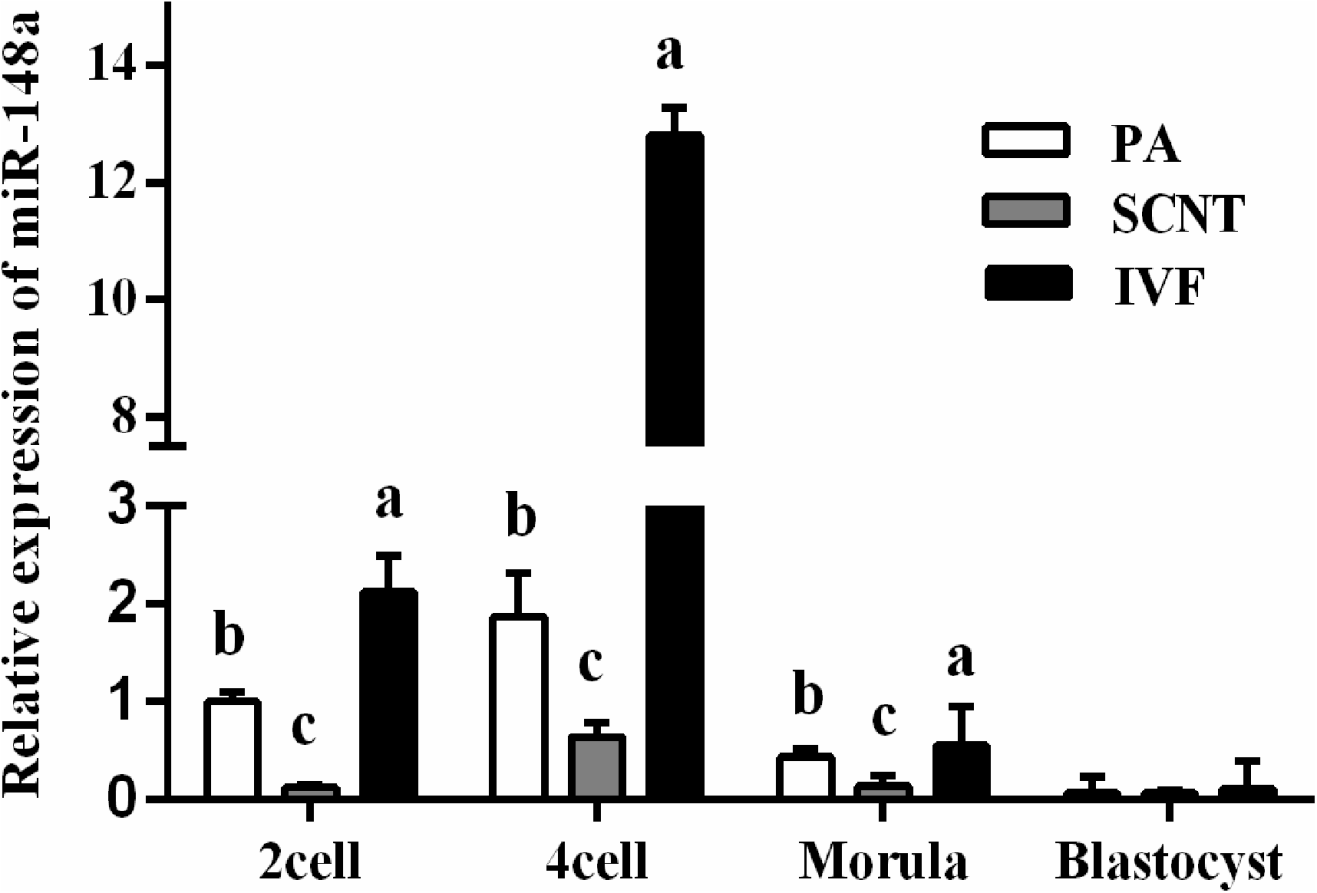
The expression pattern of miR-148a in embryos of SCNT, PA and IVF. Values with different superscript letters (a, b) within groups indicate significant difference (*p* < 0.05).

Herein, miR-148a expression was examined in SCNT, PA and IVF. The qRT-PCR results showed that miR-148a expression levels in SCNT embryos were significantly lower than in PA and IVF embryos at the 2-cells, 4-cells and morula stages (*P* < 0.05; Fig 4.). However, no significant differences were noted in the blastocyst stage (*P* > 0.05).

### Effect of miR-148a overexpression on SCNT embryo development

To examine the effect of miRNA-148a on the developmental capacity of bovine SCNT embryos, miR-148a overexpressing donors were used to produce nuclear transfer embryos. Developmental rates of the cleavage and blastocyst stages in SCNT embryos were analyzed from 3 replicates (Table 2). All SCNT embryos showed similar cleavage rates ranging from approximately 70.7% to 75.2%. The rates of the miR-148a-NT group were significantly higher than those of the con-NT group (*P* < 0.05), with no significant differences noted between the miR-con-NT and con-NT groups (*P* > 0.05). Furthermore, the rate of the blastocyst stage was significantly higher in the miR-148a-NT group relative to the con-NT and miR-con-NT groups (*P* < 0.05). Moreover, a significantly higher total cell number (*P* < 0.05) was observed in the miR-148a-NT (39.15±3.9) derived embryos relative to the con-NT group and miR-con-NT group (33.25±7.14, 34.38±8.50).

**Table 2:** Effect of miR-148a on the development of porcine SCNT embryos.

Treatment	No. of cultured embryos (replicates)	No.of cleaved embryos (% ± S.E)	No.of blastocysts (% ± S.E)	Mean±S.E of cells in blastocyst(n = 4)
con-NT	181(3)	128(70.7 ± 2.02)^a^	26(14.36 ± 2.56)^a^	33.25 ± 7.14^a^
miR-148a-NT	226(3)	170(75.2 ± 1.87)^b^	47(20.79 ± 1.84)^b^	39.15 ± 6.36^b^
miR-con-NT	194(3)	141(72.68 ± 1.48)^a^^b^	29(14.90 ± 1.48)^a^	34.38 ± 8.50^a^

^ab^Values with different superscripts in the same column are significantly different (*p* < 0.05). SEM, standard error of the mean.

### Effects of miR-148a overexpression on SCNT embryo DNA methylation and histone modification

**Fig 5:**
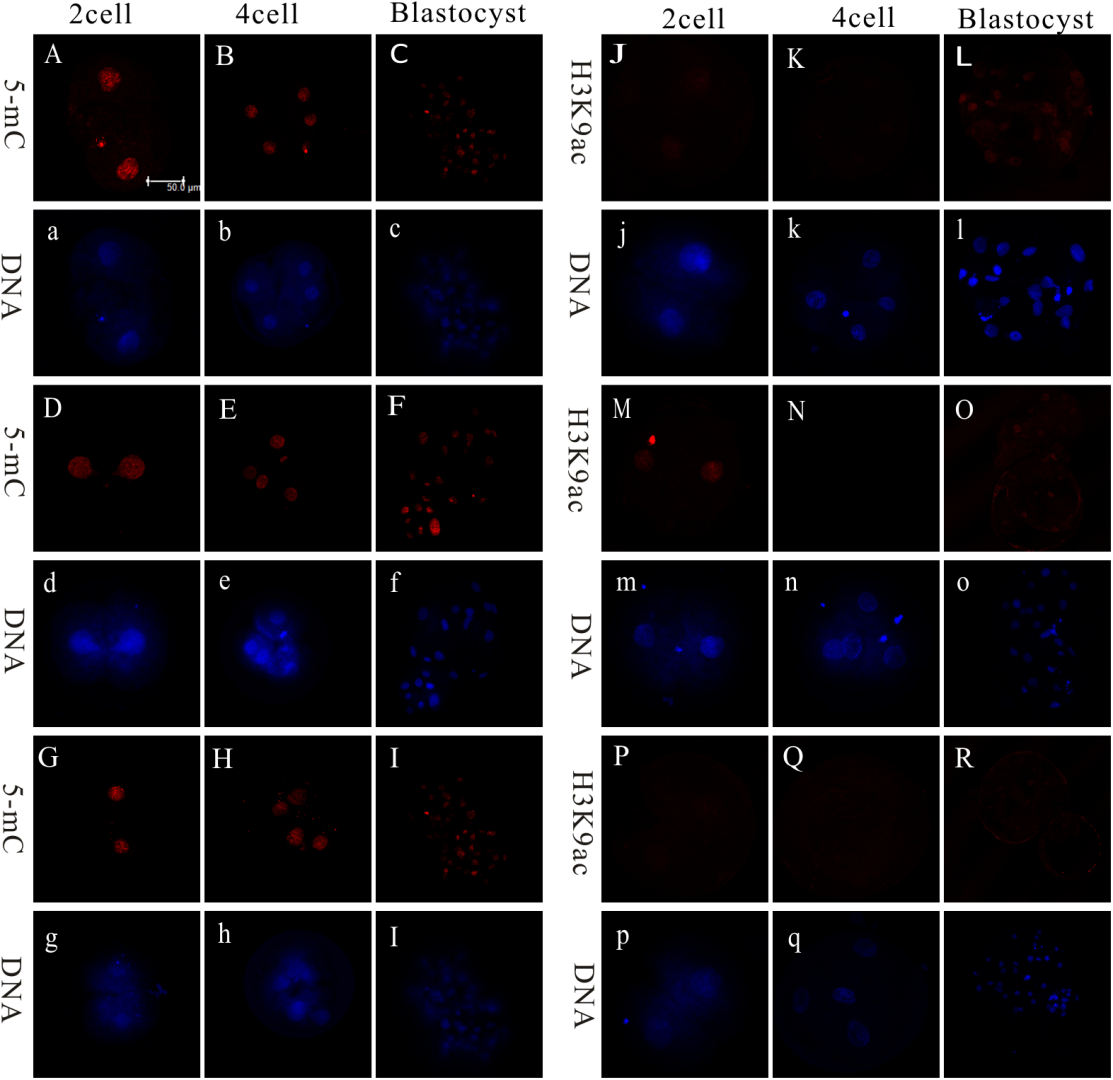
Expression patterns of 5-mC and H3K9ac in porcine Con-NT embryos, miR-148a-NT embryos, and miR-con-NT embryos. Note: A-C, J-L: Con-NT; D-F, M-O: miR-148a-NT; G-I, P-R: miR-Con-NT; A-I: 5-mC, J-R: H3K9ac; Red: 5-mC or H5K9ac; Blue: DNA; 5-mC and H3K9ac (capital letter); DNA (lowercase letter). Scare bar = 50μm.

**Fig 6:**
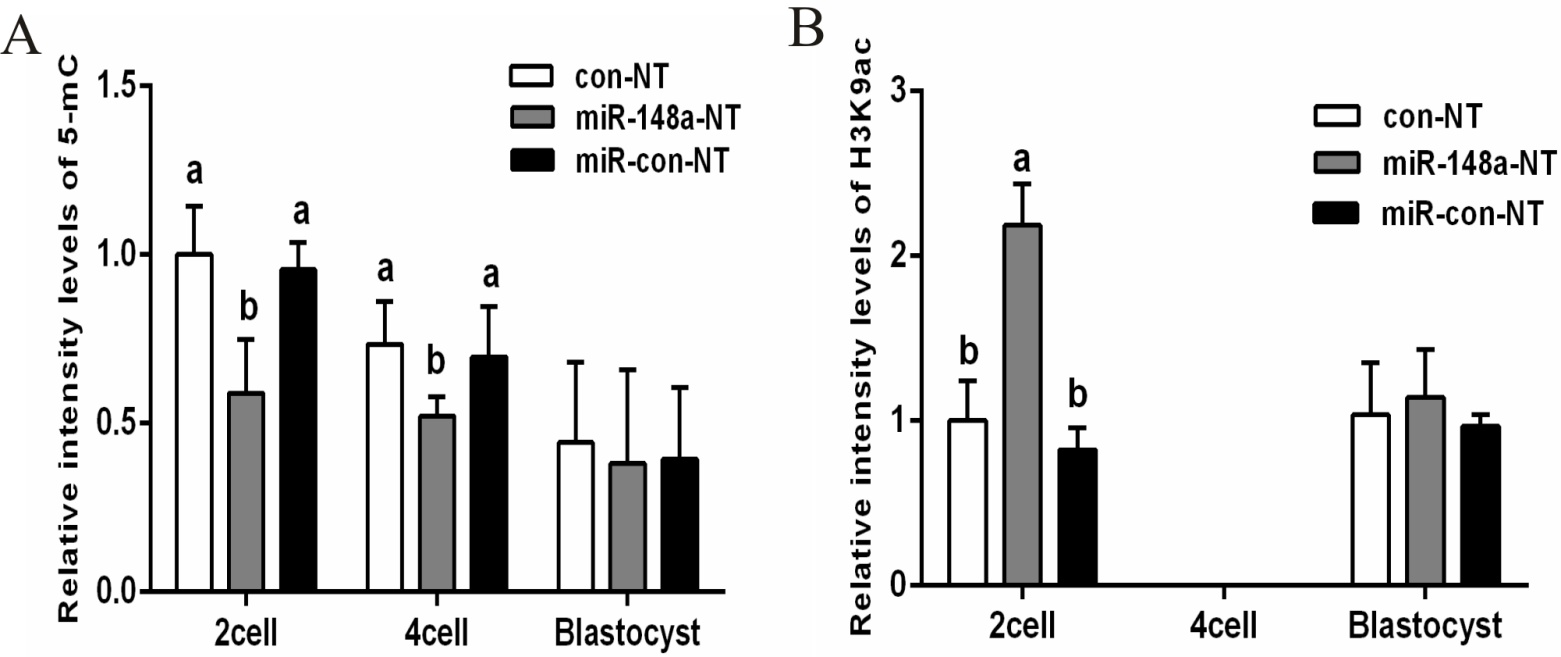
**Relative levels of 5-mC (A), H3K9ac (B) in porcine SCNT embryos.** Values with different superscript letters (a, b) within groups indicate significant difference (*p* < 0.05).

DNA methylation levels and histone modifications were examined during the 2-cell, 4-cell, and blastocyst stages via immunohistochemistry, with H3K9ac fluorescence also measured (Fig 5). As shown in Fig 5, the relative fluorescence intensity of DNA methylation in the 2-cell and 4-cell stages was significantly lower in the miR-148a-NT group relative to the con-NT or miR-con-NT groups (*P* < 0.05). However, no differences in DNA methylation levels were observed during the blastocyst stages. When examining the relative fluorescence intensity of H3K9ac during the 2-cell stage, the miR-148a-NT group showed significantly higher levels than did the con-NT or miR-con-NT groups (*P* < 0.05; Fig 6). However, no fluorescence was detected at the 4-cell stage and no differences were observed during the blastocyst stage (*P* > 0.05).

### Effects of miR-148a overexpression on pluripotent genes

**Fig 7:**
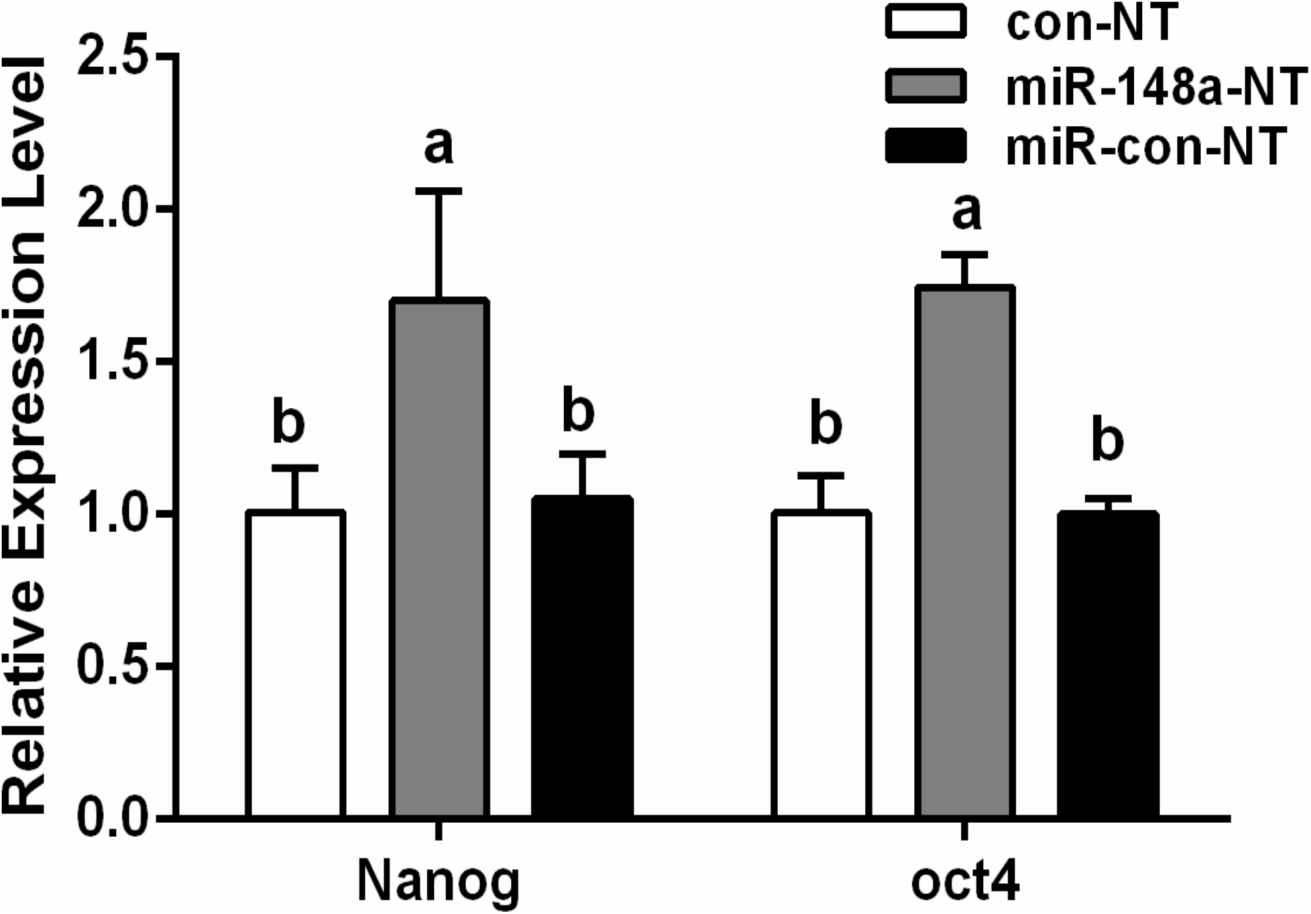
Relative expression of Oct4 and Nanog genes in porcine SCNT embryos at Blastoyst stages. Values with different superscript letters (a, b) within groups indicate significant difference (*p* < 0.05).

When examining the pluripotent genes *Oct4* and *Nanog*, the miR-148a-NT group showed significantly higher expression levels than did the con-NT or miR-con-NT groups (*P* < 0.05), with no significant differences noted between the con-NT and miR-con-NT groups (*P* > 0.05; Fig 7). These results show that miR-148a overexpression can improve *Oct4* and *Nanog* expression levels, thereby increasing the embryo developmental potential.

## Discussion

DNA methylation is very important for normal embryonic development and gene expression. In differentiated somatic cell nuclei, methylation levels are high [3, 24]. In order to obtain a high developmental competence, methylation levels must be reduced to those of a totipotent embryonic state [25]. DNA methylation of CpG dinucleotides is mediated by DNMTs. In SCNT-derived embryos, *DNMT1* is aberrantly expressed and mainly acts as a maintenance methyltransferase, which may contribute to the aberrant methylation status [26]. In previous studies, *DNMT1* knockdown has been shown to decreased DNA hypermethylation levels carried by donor cells in NT embryos [12]. Furthermore, previous studies have indicated that using donor cells with low *DNMT1* transcript levels could improve embryonic developmental competence relative to donor cells with high *DNMT1* transcript levels. Collectively, these findings suggest that the developmental competence of NT embryos may be improved by reducing donor nuclei hypermethylation.

In recent studies, aberrant *DNMT1* expression has been shown to induce miR-148a silencing and *DNMT1* has been shown to be a target of miR-148a in several tumor types [27]. Therefore, miRNA-148a upregulation reduces *DNMT1* expression. In this study, *DNMT1* was predicted to be targeted by miR-148a using TargetScan, miRanda, and DIANA-microT and then confirmed in porcine PFF cells using a luciferase reporter assay. Additionally, miR-148a expression was measured in SCNT porcine embryos at various developmental stages and showed that miR-148a expression is lower in SCNT embryos relative to IVF and PA embryos. These results indicate that aberrant *DNMT1* expression in SCNT embryos could be attributed to their reduced miR-148a expression and that miRNA-148a can serve as a candidate to modulate nuclear reprogramming in porcine embryos.

In other studies, RNAi-mediated *DNMT1* knockdown was found not only significantly decreasing DNA demethylation level, but also significantly increasing the rate of cleavage and blastocyst formation of bovine SCNT embryos [26]. Herein, over-expression of miR-148a reduced the DNMT1 expression of fibroblasts, subsequently facilitated the removal of the DNA hypermethylation pattern of donor cells, let the epigenetic status of cells to become similar to that of a blastomere. Using hypomethylation cells as the donor of SCNT might improve the development competence of SCNT embryos. These results showed that miR-148a not only reduced DNA methylation levels, but also improved embryonic development and ultimately SCNT embryo success. To our knowledge, this is the first examination of the effect of using porcine donor cells overexpressing miR-148a.

Furthermore, this study showed that miR-148a overexpression increases H3K9ac levels during the 2-cell stage in SCNT embryos. DNA methylation and histone modifications are 2 important epigenetic modifications that occur during animal embryonic development and subsequently modulate gene expression [28]. Previous studies have shown that aberrant DNA methylation and histone acetylation in SCNT embryos inhibits gene transcription [11]. A study by Enright found that treating the donor cells with 5-aza-dc could not only reduce DNA methylation levels, but could also restore histone H3 acetylation levels in NT embryos to that of IVF embryos [29]. Moreover, DNMTs have been shown to combine with methyl-CpG-binding protein (MeCP2) to recruit histone deacetylases (HDACs), which results in a reduction of acetylation [30]. Therefore we assumed that the effect of miR-148a on histone acetylation was most likely indirect.

Previous studies have shown that pluripotent gene expression was correlated with the developmental efficiency of SCNT embryos, and the reactivation of pluripotent genes could enhance the reprogramming efficiency [31]. The epigenetic modification agent 5-aza-dC or TSA improved the expression patterns of pluripotent transcription factors (*Oct4*, *Nanog*) resulting in high development of cloned embryos [32–34]. In this study, the pluripotent markers *Oct4* and *Nanog* were examined and shown to have higher expression levels in the miR-148a-NT group relative to the miR-con-NT or con-NT groups. This result shows that miR-148a overexpression in donor cells can reactivate pluripotent genes, thus contributing to a higher embryo developmental competence. Lopez-Bertoni’s research has shown that coexpressing *Oct4* and *Sox2* could downregulate miR-148a via DNA methylation [35]. While the specific mechanism by which miR-148a contributes to the restoration of pluripotent gene expression in cloned embryos was not been examined, it would seem likely that it is attributed to a reduction of methylation in the promoter regions of these genes.

In summary, miR-148a overexpression can inhibit *DNMT1* expression and promote DNA hypomethylation in PFF cells and SCNT embryos. To the best of our knowledge, this is the first report on the regulation of cellular methylation status by miRNAs in PFFs, and the effects of miRNA on development of SCNT embryos.
